# Supplementary material for: Effects of prolonged continuous computer gaming on physical and ocular symptoms and binocular vision functions in young healthy individuals
Source: PeerJ. 2019 Jun 4;7:e7050. doi: 10.7717/peerj.7050 (PMC6555390; doi:10.7717/peerj.7050)
Supplement: Supplemental Information 1 [file peerj-07-7050-s002.zip › Questionnaire.pdf]

Korean Version

설 문 지

피검자 코드:____________________ 나이:__________ 성별: 남자/여자

날짜:____________________________

이 설문지는 신체적 또는 안구 증상을 불편함에 대한 간단한 설문입니다.
각 증상에 대해 가장 알맞은 항을 체크(✓)하시기 바랍니다.

신체 증상

	없다	조금 있다	보통이다	그렇다	매우 그렇다
1. 어깨가 아프다					
2. 목이 아프다					
3. 등이 아프다					
4. 두통					

안구 증상

	없다	조금 있다	보통이다	그렇다	매우 그렇다
1. 눈이 충혈 된다					
2. 눈에 압박감이 있다					
3. 눈이 가렵다					
4. 눈이 피로하다					
5. 눈이 건조하다					
6. 눈물이 난다					
7. 눈이 따가웠다					
8. 물체가 흐려 보인다					
9. 눈이 아프다					

English version

Questionnaire

Subjects Code:________________ Age:__________ Gender:___M/F___

Date:_________________________

This sheet is a short questionnaire with statements about discomforts which involve your physical and ocular symptoms.

Check the column that best represents the occurrence of each symptom.

Physical symptoms

	None	Slight	Moderate	Severe	Very severe
1. Shoulder pain					
2. Neck pain					
3. Back pain					
4. Headache					

Ocular symptoms

	None	Slight	Moderate	Severe	Very severe
1. Red eyes					
2. Eyestrain					
3. Itchy eyes					
4. Tired eyes					
5. Dry eyes					
6. Tear eyes					
7. Irritated eyes					
8. Blurred vision					
9. Aching eyes					
